# Screen-viewing behaviours of children before and after the 2020–21 COVID-19 lockdowns in the UK: a mixed methods study

**DOI:** 10.1186/s12889-023-14976-6

**Published:** 2023-01-17

**Authors:** Ruth Salway, Robert Walker, Kate Sansum, Danielle House, Lydia Emm-Collison, Tom Reid, Katie Breheny, Joanna G. Williams, Frank de Vocht, William Hollingworth, Charlie Foster, Russell Jago

**Affiliations:** 1 Centre for Exercise, Nutrition & Health Sciences, School for Policy Studies, University of Bristol, Bristol, BS8 1TZ United Kingdom; 2 Population Health Sciences, Bristol Medical School, University of Bristol, Bristol, BS8 2PS United Kingdom; 3 Communities and Public Health, Bristol City Council, Bristol, BS1 9NE United Kingdom; 4 Applied Research Collaboration West (NIHR ARC West), The National Institute for Health Research, University Hospitals Bristol and Weston NHS Foundation Trust, Bristol, BS1 2NT United Kingdom; 5 NIHR Bristol Biomedical Research Centre, University Hospitals Bristol and Weston NHS Foundation Trust and University of Bristol, Bristol, United Kingdom

**Keywords:** Coronavirus, Screen time, Electronic device use, Sedentary behaviour, Television viewing

## Abstract

**Background:**

Restrictions during the COVID-19 pandemic have led to increased screen-viewing among children, especially during strict periods of lockdown. However, the extent to which screen-viewing patterns in UK school children have changed post lockdowns is unclear. The aim of this paper is to examine how screen-viewing changed in 10–11-year-old children over the 2020–21 COVID-19 pandemic, how this compares to before the pandemic, and the influences on screen-viewing behaviour.

**Methods:**

This is a mixed methods study with 10–11-year-olds from 50 schools in the Greater Bristol area, UK. Cross-sectional questionnaire data on minutes of weekday and weekend television (TV) viewing and total leisure screen-viewing were collected pre-COVID-19 in 2017–18 (*N* = 1,296) and again post-lockdowns in 2021 (*N* = 393). Data were modelled using Poisson mixed models, adjusted for age, gender, household education and seasonality, with interactions by gender and household education. Qualitative data were drawn from six focus groups (47 children) and 21 one-to-one parent interviews that explored screen-viewing behaviour during the pandemic and analysed using the framework method.

**Results:**

Total leisure screen-viewing was 11% (95% CI: 12%-18%) higher post-lockdown compared to pre-COVID-19 on weekdays, and 8% (95% CI: 6%-10%) on weekends, equating to around 12–15 min. TV-viewing (including streaming) was higher by 68% (95% CI: 63%-74%) on weekdays and 80% (95% CI: 75%-85%) on weekend days. Differences in both were higher for girls and children from households with lower educational attainment. Qualitative themes reflected an unavoidable increase in screen-based activities during lockdowns, the resulting habitualisation of screen-viewing post-lockdown, and the role of the parent in reducing post-2020/21 lockdown screen-viewing.

**Conclusions:**

Although screen-viewing was higher post-lockdown compared to pre-COVID-19, the high increases reported during lockdowns were not, on average, sustained post-lockdown. This may be attributed to a combination of short-term fluctuations during periods of strict restrictions, parental support in regulating post-lockdown behaviour and age-related, rather than COVID-19-specific, increases in screen-viewing. However, socio-economic differences in our sample suggest that not all families were able to break the COVID-19-related adoption of screen-viewing, and that some groups may need additional support in managing a healthy balance of screen-viewing and other activities following the lockdowns.

**Supplementary Information:**

The online version contains supplementary material available at 10.1186/s12889-023-14976-6.

## Background

Excessive screen-viewing in children is associated with a number of detrimental health outcomes including adiposity, unhealthy diet, depressive symptoms, and poorer quality of life [1], as well as reduced academic performance [2]. The majority of research has focused on television (TV) viewing, with less conclusive evidence for an association with overall screen time or non-TV screen time [1, 3]. Current advice has moved away from set thresholds for screen-viewing in favour of a more individualised approach. For example, the American Academy of Pediatrics encourage a Family Media Use Plan [4] based on a balance between screen-viewing and other activities, and the UK Chief Medical Officers recommend finding a healthy balance of physical activity and screen-viewing for the whole family [5].

Screen-viewing among children has risen over time, with a slight decrease in TV-viewing more than offset by a rise in other types of screen-viewing [6–8], especially related to new technology such as YouTube videos, social media and TV streaming services [9]. For example, total weekday screen time, estimated across 30 countries, rose between 1.3–1.4 h per day between 2002 and 2010 in 11-year-olds, with larger increases on weekends of 1.9–2.1 h [6]. More recently in the UK, total screen-viewing among 8–18-year-olds was estimated at 1.75 h more per day in 2015 than in 2000, with an average total screen-viewing of 5.4 h per day (the sum of TV, YouTube, gaming and social media screen-viewing) among 8–11-year-olds in 2019 [9]. Moreover, screen-viewing increases as children age [4, 10] by 16 min per weekday and 52 min per weekend day between ages 6 and 9 [10]. Boys typically engage in more screen-viewing, especially gaming, than girls. There are also differences by socio-economic position (SEP), with higher screen-viewing observed among lower SEP groups [3, 4, 10–13]. This could be, in part, due the home environment of children from groups with lower SEP (household income and education) facilitating screen-viewing behaviours, with greater access to media, such as TVs, in their bedroom and parents tending to watch more TV with their children [14]. Differences over time and by age are further affected by rapid changes in media usage patterns [4], with parents in 2018 reporting a wide range of reasons for children engaging in screen-viewing, including development of technological skills, entertainment, education, social interaction, and acting as a babysitter [15]. Thus, screen-viewing is complex with patterns changing over time.

After the World Health Organization declared the COVID-19 outbreak as a global pandemic in March 2020, over 100 countries introduced restrictions to reduce transmission, such as closure of schools and non-essential businesses and limitations on movement or social contact. Restrictions facilitated screen-viewing (e.g. remote learning during school closures, activity classes moved online, social contact with friends and family) and reduced the range of alternative activities (e.g. clubs, parks, social contact and active play) [16–19]. The already blurred lines between screen-viewing for entertainment, education and other purposes [4] have been blurred still further by the pandemic. A recent systematic review and meta-analysis [20] found that globally screen-viewing increased substantially during lockdowns, with primary school children most impacted, estimating that they engaged in 1.04 h more leisure-time screen-viewing per day, rising to 1.39 h more when school work was included. Evidence for differences by SEP are limited, but one study found associations between financial stress due to COVID-19 and increased children’s screen-viewing [21]. Thus, the COVID-19 pandemic has led to increased screen-viewing for children, especially during periods of strict lockdowns and school closures, and already disadvantaged groups may have been impacted differentially.

A key limitation in the understanding of how screen-viewing patterns have changed as a result of lockdowns is the methods used to recruit the sample, with most participants recruited via social media during the most restrictive phases of the various lockdowns [20]. There was high heterogeneity in estimates of screen-viewing across different studies undertaken under different levels of restrictions [20] and levels of screen-viewing changed with the level of restrictions [22], suggesting that much of the observed increase might be attributable to short term fluctuations due to COVID-19 restrictions. However, many adolescents reported struggling to return to their pre-pandemic behaviours [22]. During a period of eased restrictions where schools were open in Switzerland, screen-viewing remained relatively stable both within person and over time [23] suggesting little change in screen-viewing habits without external pressure. Furthermore, changes over the 1.5 years of the pandemic may be further complicated by longitudinal age-related change over this time, with steeper increases in screen-viewing in primary school children during the COVID-19 pandemic [21] than seen before [10].

The aim of this paper is to examine how screen-viewing changed in 10–11-year-old children over the COVID-19 pandemic in the UK, how this compares to before the pandemic, and what are the influences on screen-viewing behaviour. Specifically, we use a mixed methods approach to provide a fuller more in-depth picture, comparing quantitative data on longitudinal screen-viewing before the pandemic and cross-sectional screen-viewing pre- and post-2020/21 lockdown, and using qualitative data to explore perceptions of and reasons for changes in screen-viewing at different stages of the pandemic.

## Methods

Data are from the Active-6 study [24], designed to collect new data post 2021 lockdown for comparison with pre-COVID-19 data from the B-Proact1v study [25]. Full details of both projects are described elsewhere, but briefly, B-Proact1v was a longitudinal study of primary school children aged 5–11 years and their parents/carers, recruited from 57 schools in the southwest of England. This paper reports data from 1223 children from 47 schools collected between March 2015 and July 2016, when they were aged 8–9 years, and 1296 from 50 schools between March 2017 and May 2018, when they were aged 10–11 years. In Active-6 between May and December 2021, 23 of the same schools that took part in B-Proact1v in 2017/18 were revisited, and quantitative data collected from 393 children aged 10–11 and a parent/carer, as well as qualitative data from children and parents between August and September 2021. In this paper, we report pre-COVID-19 longitudinal change between ages 8–9 and 10–11, cross-sectional comparisons for children aged 10–11 between pre-COVID-19 and post 2020/21 lockdowns, and qualitative perspectives on the COVID-19 period between March 2020 and Autumn 2021. Both studies received ethical approval from the School of Policy Studies Ethics Committee at the University of Bristol, UK, and all participants provided informed consent/assent for both qualitative and quantitative aspects of this study [26]. All methods were carried out in accordance with relevant guidelines.

### Quantitative data

Both studies included a child and a parent/carer questionnaire. Parents/carers were asked about the number of hours their child typically spent in screen-viewing activities on weekdays and at weekends. As the questions differed between the two studies (see Additional File 1; Table S1 for details) we derived average minutes per day spent TV-viewing (including on-demand and streaming services) and total minutes of screen-viewing (including TV) for each study as follows. In the pre-COVID-19 study, separate questions were asked about time spent on TV-viewing (including on-demand and streaming services), computers, phones/tablets and games consoles and time spent multiscreen-viewing (i.e. using multiple devices simultaneously), each coded from ‘None’ to ‘4 h or more’. We converted these to minutes by taking the midpoints of each category. For total minutes of screen-viewing (including TV), we summed the minutes for TV, computers, phones/tablets and games consoles, and subtracted the minutes of multiscreen-viewing. In the post-lockdown study, parents/carers were asked to report both TV-viewing (including streaming) and total leisure screen-viewing (including TV) in hourly categories for ‘Less than 1 h’ up to ‘ > 5 h’. TV-viewing was re-coded to combine the upper categories (4–5 h and > 5 h) for consistency with the pre-COVID-19 scale. For both variables, the midpoints were taken to calculate average minutes of TV-viewing and average total leisure screen-viewing (including TV). In the post-lockdown study only, we also asked how many hours their child spent screen-viewing for schoolwork during the week, on the same hourly scale, and converted to minutes by taking midpoints. Note that due to the changes in the format of the questions, differences in total screen-viewing should be treated with caution, as this process is likely to overestimate screen-viewing in the pre-COVID-19 sample, and under-estimate in the post-lockdown sample.

Children reported their access to different screen-viewing devices: PC or laptop, mobile phone or tablet, and games console (TV or handheld). Pre-COVID-19, we recoded responses ‘Own a device kept in the bedroom’, ‘Own a device not kept in the bedroom’ and ‘Shared device in the home’ to indicate access to a given device, compared to ‘Device in the home but don’t use it’ and ‘Do not have device in home’. Post-lockdown, we simply asked whether they had access to each device. Note, we did not collect access to a TV in 2021, as pre-COVID-19 over 99% of children had access. The number of these types of devices that the child reported they had access to was also calculated.

Parents were asked to report their child’s date of birth, gender and the highest education qualification in the household. Highest education was recoded as ‘Up to A level (exam at age 18) or equivalent’ and ‘University degree (or equivalent) or higher’. Deciles of Index of Multiple Deprivation [27] (IMD) were determined based on home postcode, with lower deciles indicating a greater level of deprivation.

### Qualitative data

The qualitative phases of the Active-6 project are explained in detail elsewhere [28]. In brief, twenty-one parents, all of whom were mothers, took part in one-to-one semi-structured interviews between September and December 2021, and 47 children from six schools took part in six focus groups in December 2021. The interviews and focus groups were conducted as part of a larger project (Active-6) that explored children’s physical activity and screen viewing at different stages on the COVID-19 pandemic. The present study draws upon qualitative data from these discussions concerned with children’s screen viewing behaviour and home-based activities. Separate topic guides were developed for each participant group and can be seen in Additional File 2.

All interview and focus group participants had participated in the quantitative aspects of the Active-6 project. Child focus group and parent interview recruitment processes were independent. Convenience sampling was used to recruit parents due to recruitment challenges, whilst purposive sampling was used to recruit children, to ensure even ratios of gender, accelerometer-measured activity levels, and school area SEP (IMD determined from school post code).

### Data analysis

We used a partially mixed concurrent equal status mixed-methods design, with 2021 qualitative and quantitative data collection and analyses occurring in parallel at approximately the same time but findings not combined until the final interpretation [29]. This approach was adopted due to the larger study design which focused on physical activity behaviour, with qualitative and quantitative data collected over the same time period but capturing different aspects of the COVID-19 pandemic.

#### Quantitative analysis

We reported descriptive summaries of demographic and screen-viewing variables for ages 8–9 and 10–11 years pre-COVID-19 and for ages 10–11 post 2020/21 lockdown and for the qualitative sub-samples. To aid interpretation, the screen-viewing variables are reported in summaries as continuous variables, via mean and standard deviation, and as median and inter-quartile range due to the skewed nature of the data. We also reported missing data in the quantitative data.

To compare pre-COVID-19 and post 2020/21 lockdown screen-viewing in 10–11-year-olds, we modelled minutes of TV and total screen-viewing with Poisson mixed models to account for the underlying discreteness of the data, with children nested within schools to reflect the study design. We reported relative risks (RR), which can be interpreted as a multiplicative effect on the outcome associated with the post-lockdown time point. All models were adjusted for age, gender, household education, seasonality (using second order harmonic sine/cosine functions [30]), and differences in COVID-19 restrictions at the time of data collection [24]. We also tested interactions between time period (pre -COVID-19/post-lockdown) and gender, and time period and household education, to see if post-lockdown differences varied by gender and household education. It was not possible to stratify data due to the limited sample size.

As screen-viewing depends on access to screens [10], we reran the screen-viewing models adjusting for number of types of devices in a sensitivity analysis. All quantitative analysis was undertaken in Stata v17 [31] and MLwiN [32], using the Stata command runmlwin [33].

#### Qualitative analysis

The framework method was used to support qualitative data analysis [34]. This consisted of seven stages: 1) verbatim transcription by a university approved transcription service; 2) data familiarisation; 3) coding; 4) developing a working analytical framework; 5) applying the analytical framework; 6) charting data into the framework matrix; and 7) interpreting the data. In the third stage, two transcripts from both participant groups were independently coded by three researchers (parent interviews: RW, BT, and TR; child focus groups: RW, DH, and KS) using a mixture of inductive and deductive codes. Interview content and interpretations were discussed amongst the researchers and separate codebooks for parents and children were developed. RW then applied these codebooks to the remaining transcripts. We performed independent coding to facilitate researcher reflexivity and support a more nuanced and deeper interpretation of the data.

Verbatim quotes are used to illustrate the qualitative analytical narrative and interpretation of data. Parent quotes are presented alongside the gender of the parent and their child and IMD decile of their home postcode (lower deciles indicate more deprived areas), while child focus group quotes are presented alongside the child’s gender. Due to difficulties identifying individual children during transcription of focus groups, IMD decile of individual children is not provided.

## Results

### Quantitative results

**Fig. 1:**
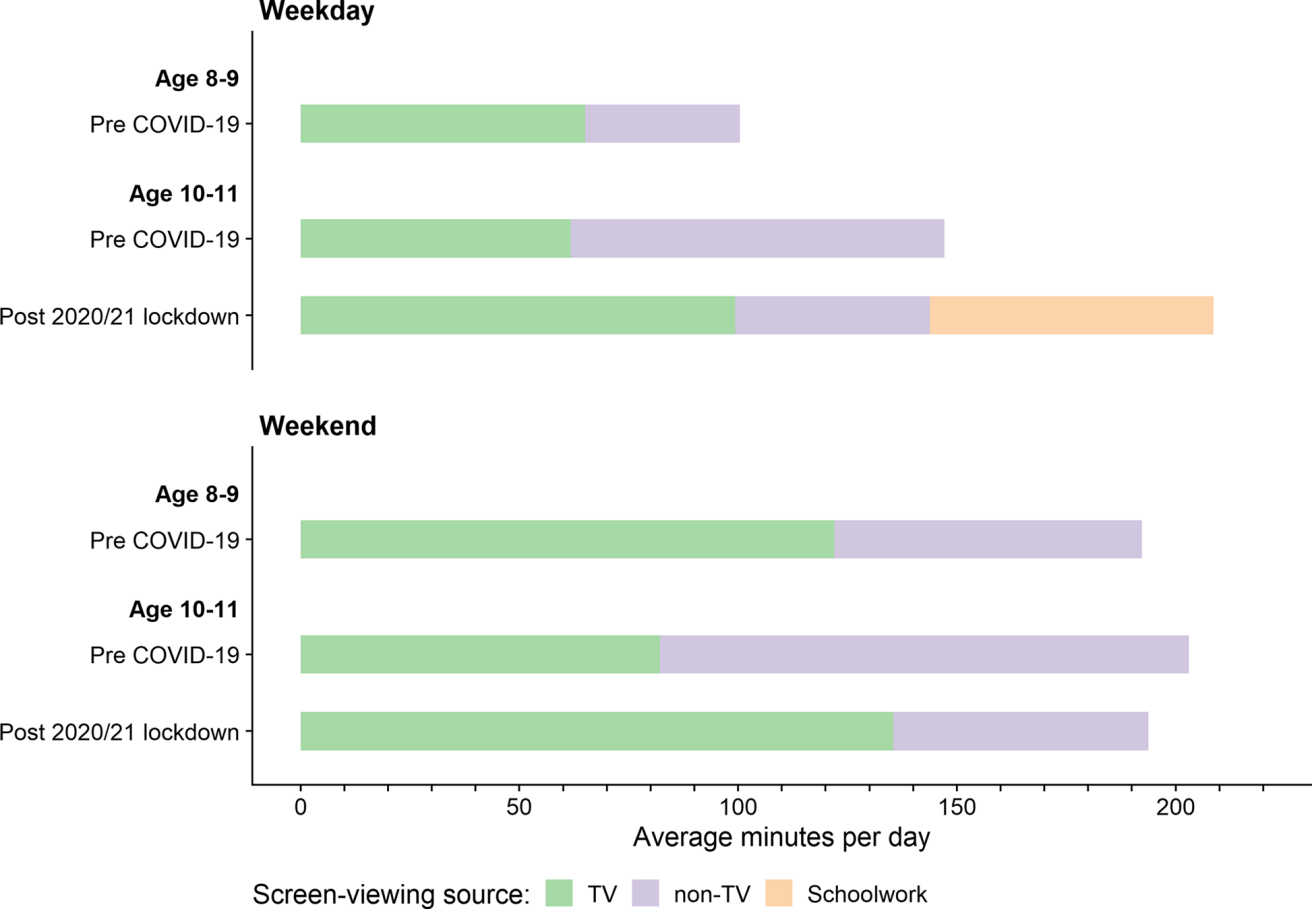
Mean minutes children spend engaged in types of screen-viewing activities pre-COVID-19 and post 2020/21 lockdown. Note that the amount of time children spent on screens for schoolwork during the week was only measured post-lockdown

Missing data (Table S2) was between 1–19% pre-COVID-19 (2017/18) and 1–9% post-lockdown (2021), with the majority of missing data due to missing questionnaires. In the pre-COVID-19 sample, total weekday leisure screen-viewing increased by 47 min between ages 9 and 11, while TV-viewing remained the same (Table S3, Fig. 1). On weekends, total screen-viewing remained the same, while weekend TV-viewing decreased. This represents an increase of approximately 50 min in non-TV screen-viewing on both weekdays and weekend days between ages 9 and 11 years. Comparing screen-viewing in 10–11-year-olds between pre-COVID-19 and post 2020/21 lockdowns, children engaged in similar total leisure screen-viewing on weekdays and weekends (144 min post lockdown compared to 147 min pre-COVID-19 on weekdays, and 203 min vs. 194 min on weekends) but TV-viewing was higher by 37 min on weekdays and 53 min on weekends (Table S3, Fig. 1), representing an overall drop in non-TV screen-viewing of 40-60 min. We also saw a drop in access to devices between pre-COVID-19 and post-lockdown, with 99% of 11-year-olds pre-COVID-19 having access to a computer, phone, tablet or games console, compared to 94% post-COVID-19 (Table S4).

Modelled differences between pre-COVID-19 and post 2020/21 lockdown in TV-viewing and total leisure screen-viewing were adjusted for age, gender, household education, seasonality and COVID-19 restrictions. Children’s TV-viewing was higher by 68% (95% CI: 63% to 74%) on weekdays and 80% (95% CI: 75% to 85%) on weekend days in the post lockdown period (Table 1). This equates to an increase approximately equivalent to 40 min and 50 min respectively. There were differences by gender on weekdays, with the difference for girls 10% (95% CI: 8% to 13%) higher than for boys, and by household education on both weekdays and weekends, with the difference among children from households with lower educational attainment 15% (95% CI: 12% to 18%) higher on weekdays and 13% (95% CI: 10% to 15%) higher at weekends (Table 2). We also saw differences in total screen-viewing, with post-lockdown estimates 11% (95% CI: 9% to 14%; equating to approximately 15 min higher) higher post-lockdown on weekdays and 8% (95% CI: 6% to 10%; approximately 12 min higher) on weekends compared to pre-COVID-19. There were differences by gender (both weekdays and weekends) and by household education on weekends only, with the pre/post lockdown difference for girls and those from households with lower educational attainment around 3% higher (roughly equivalent to 5 min). The same patterns were seen when adjusting for number of types of devices, with household education differences slightly larger (Table S5).

**Table 1:** Modelled difference in child weekday and weekend TV-viewing between pre-COVID-19 and post 2020/21 lockdown

	Difference between pre-COVID-19 and post 2020/21 lockdown		Interaction effect	
	Relative Risk (95% CI)	*P*-value	Relative Risk (95% CI)	*P*-value
**Weekday TV-viewing**^**a**^** (mins)**		***N* = 1423**		
No interaction	1.68 (1.63 to 1.74)	< 0.0005		
Gender interaction				
Boys	1.59 (1.54 to 1.65)	< 0.0005		
Girls	1.76 (1.70 to 1.82)	< 0.0005	1.10 (1.08 to 1.13)^c^	< 0.0005
Education^b^ interaction				
up to A level	1.82 (1.75 to 1.88)	< 0.0005	1.15 (1.12 to 1.18)^d^	< 0.0005
Degree or higher	1.58 (1.53 to 1.63)	< 0.0005		
**Weekend TV-viewing**^**a**^** (mins)**		***N* = 1422**		
No interaction	1.80 (1.75 to 1.85)	< 0.0005		
Gender interaction				
Boys	1.79 (1.74 to 1.85)	< 0.0005		
Girls	1.81 (1.75 to 1.86)	< 0.0005	1.01 (0.98 to 1.03)^c^	0.609
Education^b^ interaction				
up to A level	1.93 (1.87 to 1.98)	< 0.0005	1.13 (1.10 to 1.15)^d^	< 0.0005
Degree or higher	1.71 (1.66 to 1.76)	< 0.0005		

^a^TV-viewing data includes on-demand and streaming to any device

^b^or equivalent; A level is exam at age 18

^c^RR for girls compared to boys

^d^RR for up to A level compared to degree or higher

Models are adjusted for age, gender, household education, seasonality and remote data collection

**Table 2:** Modelled difference in child weekday and weekend total screen-viewing between pre-COVID-19 and post 2020/21 lockdown

	Difference between pre-COVID-19 and post 2020/21 lockdown		Interaction effect	
	Relative Risk (95% CI)	*P*-value	Relative Risk (95% CI)	*P*-value
**Weekday total leisure screen-viewing**^**a**^** (min)**		***N* = 1420**		
No interaction	1.11 (1.09 to 1.14)	< 0.0005		
Gender interaction				
Boys	1.10 (1.07 to 1.13)	< 0.0005		
Girls	1.13 (1.10 to 1.16)	< 0.0005	1.03 (1.01 to 1.05)^c^	0.008
Education^b^ interaction				
Up to A level	1.11 (1.08 to 1.14)	< 0.0005	1.00 (0.98 to 1.02)^d^	0.928
Degree or higher	1.11 (1.08 to 1.14)	< 0.0005		
**Weekend total leisure screen-viewing**^**a**^** (min)**		***N* = 1407**		
No interaction	1.08 (1.06 to 1.10)	< 0.0005		
Gender interaction				
Boys	1.06 (1.04 to 1.08)	< 0.0005		
Girls	1.09 (1.07 to 1.12)	< 0.0005	1.03 (1.02 to 1.05)^c^	< 0.0005
Education^b^ interaction				
Up to A level	1.09 (1.07 to 1.12)	< 0.0005	1.03 (1.01 to 1.04)^d^	0.005
Degree or higher	1.07 (1.04 to 1.09)	< 0.0005		

^a^total screen-viewing includes TV-viewing

^b^or equivalent; A level is exam at age 18

^c^RR for girls compared to boys

^d^RR for up to A level compared to degree or higher

Models are adjusted for age, gender, household education, seasonality and COVID-19 restrictions

### Qualitative results

Parent interviewees tended to be slightly older and from households with higher educational qualifications than parents involved in the wider Active-6 study, and their children (who were not child focus group participants) on average tended to have lower levels of screen-time (Table S6). The children who participated in the focus groups reported similar levels of screen-viewing to children who took part in the wider study.

Three themes were generated by RW using a mixture of verbatim transcripts and summarised framework matrices of both participant groups. Themes related to children’s screen-viewing behaviour over the course of the COVID-19 pandemic between March 2020 and December 2021. These themes were: 1) Living life through a screen in lockdown; 2) Losing children to screens; and 3) Parents as the antidote to screen addiction. We constructed theme definitions as short abstracts to illustrate the scope and boundaries of the multi-faceted central organising concept of each theme that can be seen in Table 3. All themes were reflected within data within both participant groups.

**Table 3:** Definitions of qualitative themes

	Theme name	Theme definition
1	Living life through a screen in lockdown	This theme explores an unavoidable increased use in screen-viewing behaviour during periods of COVID-19 lockdowns and restrictions. Unable to leave the home for large periods of the day, many aspects of parent’s and children’s lives transitioned to screen-based activities. Notably, screen-viewing activities became the medium for entertainment, socialising, education, forms of physical activity, and childcare
2	Losing children to screens	A sense of increased post-lockdown screen-viewing behaviour among children was suggested in the data. What children and parents described as ‘addiction’, stemming from increased exposure during lockdown and periods of restrictions, drew children away from activities they previously enjoyed, such as active play. However, whether this was in part also due to age-related and/or societal changes was also noted by parents
3	Parents as the antidote to screen addiction	This theme reflects the importance of the parent and their role in their child’s screen-viewing behaviour. Without limitations set by the parent, children were unable to regulate their own screen-viewing behaviour. Supporting their child’s participation in activities outside of the home constituted another way in which parents were able to break habitualised screen-viewing within the home. Yet, this required significant prioritisation due to its associated financial and time costs that not all parents and families were able to support

#### Theme 1 – Living life through a screen in lockdown

The first theme reflects an unavoidable increase in screen-viewing during periods of lockdown and restrictions. The enforcement of the first nation-wide lockdown in England in March 2020 [35] brought with it significant changes to people’s lives. Fluctuating levels of restrictions and rules limited most to their homes for large periods of the day. In what has been described as “a short-lived adventure” [28], a marked increase in feelings of boredom manifested as periods of lifestyle-limiting lockdowns were prolonged. Children expressed significant emotional challenges and a longing for fun reminiscent of their pre-lockdown lives during these periods, and screens provided one of the few opportunities for entertainment.

*“It only took me an hour to do all my [school] work. And then it was just boring the rest of the day when there was nothing to do. I just sat friends.” (Child focus group 4, child gender: male)*

*“…because you weren’t really going anywhere and you weren’t really doing anything or seeing anyone, you didn’t really feel like going out and doing anything… You didn’t really feel bothered to do anything because you couldn’t go very far… we got so sick of doing the same walks around the local area.” (Parent 10, parent gender: female, child gender: male, IMD decile 10)*

A large portion of screen-viewing enjoyment stemmed from its social elements. Online gaming, messaging applications, and video calls were noted by parents and children as popular methods of social interaction during lockdowns. A yearning to socialise with friends during periods of restrictions was vividly expressed amongst the children, with their screens providing a window to the outside world. For children without siblings, this became their only source of social interaction with similar aged children.

*“In COVID, I got quite lonely and sad because a big part of my life was, like, being around my friends, so then my mum got me more screen time because I could talk to my friends” (Child focus group 4, child gender: female)*

Some parents noted their conflicting feelings towards the social benefits and worries associated with excess screen-viewing. One parent of a single child family evoked a sense of having no other choice but to relax their screen-viewing-related rules due to their child’s social isolation in lockdown.

*“I think we worried about that [increased screen-viewing], but we also were much looser with that [screen-viewing rules] because we knew that without that, as an only child, where does he build his relationships? Where does he get his social interaction? Where does he get his outlet?” (Parent 15, parent gender: female, child gender: male, IMD decile 10)*

As schools became better-equipped and organised following the initial suddenness of school closures and prolonged periods of restrictions, teaching transitioned online during government enforced school closures. In what would have previously been a social and interactive day, parents suggested a concern at the isolated and sedentary screen-viewing necessary for their child’s education.

*“…lots of the work, especially in lockdown two, was all on the computer. It was all Google Classroom. You had to go on this, you had to watch this video on the computer, and you had to do all these activities. It was just constantly staring at a screen…” (Parent 10, parent gender: female, child gender: male, IMD decile 10)*

Physical activity was among the activities to transition online during periods of lockdown. “PE with Joe Wicks” (YouTube videos), “Just Dance”, and “Wii Sports” (video games) were commonly noted as popular activities among parents and children, providing a home-based alternative physical activity to restricted outdoor exercise.

*“…my mum set up her own rota... So before, as a wake up, we did either a few Just Dances, we’d do Joe Wicks or some yoga and then we just got on to what we need to do” (Child focus group 4, child gender: male)*

Many active clubs also made the transition online, providing live virtual training sessions.

*“…her cheerleading classes resumed online. Although it wasn’t the same, she was still able to do it towards the end.” (Parent 19, parent gender: female, child gender: female, IMD decile 10)*

Where children had previously enjoyed active play in the playground, play became mediated through electronic devices, with one parent describing this as the transition from the physical to the virtual playground.

*“His playground was an online virtual playground rather than being out with his friends.” (Parent 12, parent gender: female, child gender: male, IMD decile 8)*

Screen-viewing also became a means of occupying children, providing time and space for parents to manage other tasks, especially work, during what was a significantly challenging period. Parents spoke of an increased workload when working from home in the late 2020 and 2021 stages of the pandemic that made balancing work, home-schooling, and childcare especially challenging. This was particularly the case among families without an adult available to provide childcare throughout the day. As a result, many parents relaxed their rules and limitations on their child’s screen-viewing in order to occupy their child while they worked.

*“…I was trying to home-school and work so I wasn’t as strict about screen time because it’s occupying her. It’s all very well saying to her, “Don’t do that, do this.” but then disappearing and being on my laptop. You know, it’s quite difficult to balance those things.” (Parent 21, parent gender: female, child gender: female, IMD decile 6)*

*“And then there was also an element, if I'm honest, that when you're working and you're working full time, if it kept him quiet, that was okay. That’s an awful thing to say.” (Parent 15, parent gender: female, child gender: male, IMD decile 10)*

#### Theme 2 – losing children to screens

This theme highlights the habitualised increased screen-viewing among children following the lifting of lockdowns and COVID-19 restrictions (April – December 2021). Children and parents frequently described electronic devices as addictive. A sense of helplessness and losing children to their addictions was expressed by many parents, stemming from unavoidable use and exposure during COVID-19 lockdowns and periods of restrictions (Theme 1). A shift in attitudes amongst parents was suggested in the data where parents spoke of a need to reduce their child’s increased screen-viewing as restrictions were lifted and opportunities for non-screen-based activities returned. However, many parents described significant difficulty in reducing their child’s increased screen-viewing and what was perceived as an addiction.

*“…we'd already kind of lost [Child name] by then [Autumn 2020]… he'd gotten into a routine of coming home and watching telly and being quite content with that.” (Parent 8, parent gender: female, child gender: male, IMD decile 9)*

*“it's kind of really hard to know how we claw it [screen use] back” (Parent 7, parent gender: female, child gender: female, IMD decile 7)*

*“I think it’s [screen use] an affliction of all of them at the moment… They all want to do it.” (Parent 2, parent gender: female, child gender: female, IMD decile 5)*

*“It is very addictive, I would say… I am very addicted to it [games console]... since I got it [during lockdown]… I have spent more than 500 hours on it alone, just playing.” (Child focus group 5, child gender: male)*

Children discussed mixed feelings towards their increased screen-viewing. Although sometimes providing an enjoyable activity, children evoked feelings of sadness at how screen-viewing activities had replaced other activities they would have previously enjoyed at home. Predominantly, screen-viewing activities had replaced real-world play (i.e. imaginative, creative, and physical play that is not screen-based) where children had become accustomed to and reliant on using electronic devices, such as games consoles, for their social interaction. Feelings of guilt were suggested by children who reminisced of the enjoyment they used to experience during physical play.

*“I really loved playing on my screen because it’s something that I enjoy. But often, I do feel quite guilty, because I’m not doing stuff that I used to do quite a lot more. Because I used to always go up to my room and play games and play with all of my toys. But now I have this screen and it’s mine and I can just play with it whenever I want to, it’s kind of the only thing I ever do. I kind of just rely on it.” (Child focus group 6, child gender: female)*

This issue, however, was cyclical as children had less opportunity for real-world play with friends, with one child describing a significant decrease in children playing outside, creating an environment where children needed to use screen-viewing activities to connect with their friends.

*“Now, people don’t go outside usually, because when I go outside, I don’t usually see many people outside. It’s, kind of, boring because all the people that used to play with you, because I’ve got a park near my house and there’s usually loads of kids that live there, and I usually play with them… they still don’t go outside because they’re used to so much of the technology and watching TV, that they just forgot about everything and are just staying on there” (Child focus group 3, child gender: male)*

One parent rationalised this as screen-viewing activities were the easy option, especially during winter periods, that connects children to friends no matter where they live and without a need to leave the home.

*“it’s [screen-viewing] easy, especially now it’s darker in the evenings, it’s easy to sit in front of the telly, or for him to stay in front of a computer game or something, and not go out. (Parent 14, parent gender: female, child gender: male, IMD decile 2)*

Whether this increased use in screen-viewing was entirely due to the pandemic or was also a mixture of age-related and societal changes, were discussed by some parents. The 18-month period of restrictions brought with it many natural changes to the children. A shift to the final year of primary school coincided with changes in interests, including a transition from real-world play to screen-viewing-based play. The role of other children and peer pressures were expressed, with parents evoking the notion that children need to keep up with the technological advancements in an increasingly screen-based society.

*“Obviously with lockdown everything…being on the computer, that increased dramatically and now because she’s older again, she’s found Roblox [Multiplayer online game]. She only ever got introduced to Roblox during Year 5 because all her friends were on it… she just went on and on, “I want to play Roblox, everybody else is on it. I’m the only one that’s not on it.”… She has probably way too much screen time now just because it’s easier and obviously being older, it is difficult… she definitely has a lot more screen time than before.” (Parent 19, parent gender: female, child gender: female, IMD decile 10)*

*“…and obviously the world is becoming very much an onscreen world in lots of ways, but it does mean that they can end up spending quite a lot of time on screen...” (Parent 18, parent gender: female, child gender: female, IMD decile 4)*

#### Theme 3 – Parents as the antidote to screen addiction

The third theme describes the important role of the parent or carer in reducing the increased post-lockdown screen-viewing, perceived by both parents and children as an ‘addiction’ (Themes 1 and 2). The idea of a post-lockdown battle with their children over limiting their child’s screen-viewing behaviour was suggested by parents. The challenge and difficulties associated with limiting screen-viewing caused conflict and frustration. However, without such intervention, children would be unable to self-regulate their own screen-viewing behaviour. Parents expressed a need to be engaged and firm in the reduction of their child’s screentime. Yet, some parents felt that TV-viewing did not require limitations to the extent of more personal screen-viewing, such as smart phones, tablets, and game consoles.

*“…I have to limit it [screen-viewing] because I think otherwise she would just come home from school and be quite happy talking to her friends either on my phone or on her game.” (Parent 16, parent gender: female, child gender: female, IMD decile 10)*

*“It is still always a battle. They have a timer where they have a certain amount of time that they can go on screen-screens, but not so much telly. So things like YouTube or computer games, the iPad and stuff, then you might just sit down and watch some telly later in the evening.” (Parent 17, parent gender: female, child gender: male, IMD decile 10)*

*“[Screen-viewing has] completely rocketed. He’s a total gamer and doesn’t want to get off… I have to just say, ‘Right, that’s it’ and be very firm.” (Parent 2, parent gender: female, child gender: female, IMD decile 5)*

While screen-viewing-related rules were perceived to be an effective way of limiting the behaviour, it led to a gap in the child’s day that needed to be occupied by an alternative activity. With the end of the lockdowns came the gradual increase in activities outside of the home environment, such as school and community clubs, which offered a remedy to breaking habitualised screen-viewing behaviour. Children felt that there were few alternative fun activities to screen-viewing when at home. Despite screen-viewing not always being enjoyable, the ability to socialise and play online with friends was enough to encourage participation.

*“I do it [screen-viewing] because I just don’t want to do anything else… Nowadays when I go to football friendlies with my friends, as soon as we get back, all we do is play video games together... Half of the time it’s really boring and that’s like the only thing we can do.” (Child focus group 4, child gender: male)*

Activities outside the house, such as school and community clubs, provided a break in the routine and what had become habitualised screen-viewing behaviour within the home environment. Yet, the significant financial and time commitment associated with club participation was challenging for many parents, particularly among those from lower SEP groups, requiring a concerted prioritisation by parents in their family’s busy post-lockdown lives. Without such prioritisation and parent engagement, many children may be unable to break what has become habitualised screen-viewing at home.

*“It’s much more reduced. He likes his screen time. Don't get me wrong, but I think because all of his physical activities have kicked back in, he's out six days a week doing organised sport… That’s where all my money goes… So, I appreciate that on a lower-income family, I don’t know how they’d afford it. Because basically, all my free money pretty much goes on subs, membership. It’s a fortune… [and] it's not just an hour out of the house… it might be an hour plus all of the travelling… so it could be a three-hour session... Life has become very, very hectic with all of those activities, with work, with social life and stuff. But I think for us… we see our sports as our priority… I think that's a big thing.” (Parent 15, parent gender: female, child gender: male, IMD decile 10)*

## Discussion

The combination of quantitative and qualitative data presented in this paper provides a deeper understanding of how the screen-viewing patterns among primary school children have changed over the last two years. Parents spoke of large increases in children’s screen-viewing over the 1.5 years since the start of the pandemic, especially when COVID-19-related restrictions were at their peak, with screen-based activities seen as unavoidable, as sources of entertainment, socialising, education, forms of physical activity, and childcare. This is consistent with quantitative increases in TV and screen-viewing found during strict lockdowns [20], as well as with wider international qualitative narratives [16–19] and what researchers have described as a “necessary evil” [17]. As restrictions eased, these now established screen-based activities were described as ‘addictive’ with parents struggling to reduce and regulate their child’s screen-viewing, although this became easier for families who were able to facilitate and support alternative activities, such as school and community clubs, once they became available again. Our repeated cross-sectional quantitative data analysis indicated that weekday leisure screen-viewing among 10–11-year-olds in May-December 2021 after restrictions were lifted were 11% higher on weekdays and 8% higher on weekend days compared to 10–11-year-olds pre-COVID-19, equating to around 12–15 min difference. TV-viewing, including on-demand and streaming, increased substantially, and was 68% higher on weekdays and 80% higher on weekends post-COVID-19 lockdowns (around 40 and 50 min respectively). To our knowledge, this is the first study to report post-lockdown screen-viewing estimates in the UK.

The quantitative estimates are lower than perceived increases described by parents in the qualitative interviews. This may in part be due to changes in the questions asked, which will result in a slight underestimate of the difference in total screen-viewing between pre-COVID-19 and post-lockdown. However, we note also that these perceptions of COVID-19 changes in screen-viewing occur against a background of a secular trend of increasing screen-viewing, which are further complicated by the simultaneous longitudinal age-related changes. Screen-viewing increases with age [4, 10, 11], and the final years of primary school are particularly subject to change, with smartphone ownership in the UK doubling between the ages of 9 and 10 in 2019 [9]. The pre-COVID-19 longitudinal data reported in this paper showed that weekday leisure screen-viewing increased by around 50 min between ages 9 and 11, a much steeper increase than the 16 min we previously reported between ages 6 and 9 [10]. Thus, parent perspectives of increases in screen-viewing since the start of the pandemic may be due to the usual age-related changes over this time, rather than attributable to the COVID-19 pandemic. This was also expressed by parents, as their child’s interests changed as they got older, with the final year of primary school coinciding with many other life changes. Note that this difficulty in separating self-reported changes into those due to COVID-19 and those from longitudinal change is particularly an issue in studies of screen-viewing during the pandemic, as the majority rely on retrospective measures of perceived change in screen-viewing. Our findings highlight that care should be taken in interpreting studies that use self-reported change in screen-viewing since the start of the pandemic, as longitudinal age-related changes may skew results.

Compared to the small pre- and post- lockdown differences in total leisure screen-viewing, TV-viewing rose sharply post-COVID-19 lockdowns by around 70–80%. Thus, non-TV-viewing, such as social media and gaming, seems to have reduced. This contrasts with the slow decline in TV-viewing observed over the last twenty years before the pandemic [6]. Pre-pandemic, changes in technology have led to changing patterns in types of screen-viewing [4, 7–9], especially an increase in non-TV screen-viewing such as social media and gaming. However, the COVID-19 pandemic appears to have changed this balance. Moreover, unlike short-term increases in screen-viewing during lockdowns, TV-viewing remained high once restrictions were lifted, which may reflect a longer-term change in patterns and types of screen-viewing. Parent interviewees imposed fewer rules and restrictions around TV-viewing than tablets or computer games, which may reflect a differentiation between perceptions of ‘acceptable’ and ‘unacceptable’ types of screen-viewing as technology continues to evolve. In other research, parents have described TV-viewing as a relaxation tool that was apparent in their own childhood, suggesting it as a more normalised type of screen viewing that they may not limit in the same way as newer forms of screen-viewing [36]. Although not directly asked in our pre-COVID-19 comparator group, screen-viewing for school work was estimated at an average of 65 min per day post- lockdowns, broadly aligning with estimates during lockdowns from recent meta-analysis [20].

Qualitative data suggested that children’s screen-viewing was higher during strict COVID-19 restrictions than estimated post-lockdown. As restrictions eased, parents highlighted the importance and difficulty of imposing rules to help manage their children’s screen-viewing. Previous evidence has found that parental restrictions are associated with lower screen-viewing in children [10, 37], including during the COVID-19 pandemic [20, 21], which could partially explain why post-lockdown average screen-viewing was not as high as reported in other studies during lockdowns [20]. The importance of the parent in ‘striking a balance’ between screen-viewing and other activities has been highlighted in pre-pandemic qualitative research among similar-aged children [38]. However, parents spoke of additional challenges in reimposing rules after they had been relaxed during periods of strict lockdown, often characterised as a ‘battle’ that required a firm parenting approach. Furthermore, while screen-viewing-related rules were perceived to be an effective way of limiting behaviour, it left a gap in the child’s day that needed to be filled with alterative enjoyable activities, such as school and community clubs, as many children perceived a lack of enjoyable non-screen-based activities within the home. Facilitating alternative activities required significant financial and logistical support from parents, that may not be feasible for some, and might be particularly challenging for lower SEP groups [39]. We found higher levels of post-COVID-19 lockdown TV and total screen-viewing among children from households with lower educational qualifications, while another study found higher screen-viewing when parents reported financial stress due to the pandemic [21]. Lower SEP is generally associated with higher levels of screen-viewing [4, 10, 11], so children with higher levels of pre-pandemic screen-viewing who have increased during lockdowns may be at risk of maintaining these high levels post-pandemic if their parents lack the resources to facilitate alternative activities for their child.

### Strengths and limitations

This study has a number of strengths. It uses quantitative data collected at each timepoint, rather than retrospectively, and the qualitative data draws on both child and parent perspectives. The combination of these data allows us to explore changes over the course of the pandemic and afterwards, and compare these with pre-COVID-19 patterns. This gives a unique insight into a complex story. Our TV-viewing variable includes on-demand and streaming to other devices, both of which are increasingly common ways for children to watch TV [9]. However, changes in the screen-viewing questions mean that the estimate of the difference in total screen-viewing is likely to be slightly underestimated and so these represent conservative estimates, although differences between gender and education categories are more robust. The evidence suggests that patterns of screen-viewing may have changed as a result of the pandemic, but as we did not collect individual types of screen-viewing apart from TV, we are unable to comment on whether individual types of screen-viewing such as gaming or smartphone/tablet use have changed. We also did not collect data on screen-viewing for school work pre-pandemic. Furthermore, it is important to note that findings were drawn from a UK context and require further exploration in a wider variety of international settings. Finally, parents and children from higher SEP groups were more likely to take part in both the quantitative and qualitative studies, with the majority of parents in the quantitative data and all of the parent interviewees being mothers. Thus, while we have identified lower SEP children as being potentially at risk, we have limited ability to explore this in much detail and so this is an area that warrants further exploration.

## Conclusion

Although screen-viewing was higher post-lockdown compared to pre-COVID-19, the high increases reported in previous studies have not, on average, been sustained post-lockdown, and may be attributed to a combination of short-term fluctuations during periods of strict restrictions, parental support in regulating post-lockdown behaviour, and age-related rather than COVID-19 specific increases in screen-viewing. However, types of screen-viewing have changed post-COVID-19 lockdowns, in particular an increase in TV-viewing. Moreover, socio-economic differences in our sample suggest that not all families were able to break the COVID-19-related habitualisation of screen-viewing, and that some groups may need additional support in managing a healthy balance of screen-viewing and other activities following the COVID-19 pandemic lockdowns.

## Supplementary Information

**Additional file 1: Table S1. **Summary of screen-viewing questions asked in each survey.** Table S2. **Summary of missing data.** Table S3. **Time spent in screen viewing (mins) by child gender and household education.** Table S4. **Access to different devices pre and post-COVID-19.** Table S5. **Modelled difference in child weekday and weekend total leisure time screen viewing between pre- and post-COVID-19, adjusting for number of types of devices.** Table S6. **Post-COVID demographic and screen-viewing of interview and focus group participants compared to quantitative sample.

**Additional file 2:** **Supplementary File.** Interview and focus group topic guides.

## Data Availability

As the Active-6 project is still ongoing, data are not currently available. Data will be made available in the University of Bristol’s data repository at the end of the project (https://data.bris.ac.uk/data/).

## Abbreviations

SEP: Socioeconomic position
TV: Television
