# Supplementary material for: Screen-viewing behaviours of children before and after the 2020–21 COVID-19 lockdowns in the UK: a mixed methods study
Source: BMC Public Health. 2023 Jan 17;23:116. doi: 10.1186/s12889-023-14976-6 (PMC9843116; doi:10.1186/s12889-023-14976-6)
Supplement: Supplementary file 1 — Additional file 1: Table S1. Summary of screen-viewing questions asked in each survey. Table S2. Summary of missing data. Table S3. Time spent in screen viewing (mins) by child gender and household education. Table S4. Access to different devices pre and post-COVID-19. Table S5. Modelled difference in child weekday and weekend total leisure time screen viewing between pre- and post-COVID-19, adjusting for number of types of devices. Table S6. Post-COVID demographic and screen-viewing of interview and focus group participants compared to quantitative sample. [file 12889_2023_14976_MOESM1_ESM.pdf]

Screen-viewing behaviours of children before and after the 2020-21 COVID-19 lockdowns in the UK: a mixed methods study

Authors: Ruth Salway, Robert Walker, Kate Sansum, Danielle House, Lydia Emm-Collison, Tom Reid, Katie Breheny, Joanna G Williams, Frank de Vocht, William Hollingworth, Charlie Foster and Russell Jago

Supplementary Tables

Table S1: Summary of screen-viewing questions asked in each survey

Table S2: Summary of missing data

Table S3: Time spent in screen viewing (mins) by child gender and household education

Table S4: Access to different devices pre and post-COVID-19

Table S5: Modelled difference in child weekday and weekend total leisure time screen viewing between pre- and post-COVID-19, adjusting for number of types of devices

Table S6: Post-COVID demographic and screen-viewing of interview and focus group participants compared to quantitative sample

Table S1: Summary of screen-viewing questions asked in each survey

<table>
<tr><th>B-Proact1v
(pre-COVID-19)</th><th>Active-6
(post 2020/21 lockdown)</th></tr>
<tr><td colspan="2">Total Screen-viewing time (asked for weekdays and weekends separately)</td></tr>
<tr><td>On a weekday/weekend, how many hours does your child spend watching programmes on TV (including DVDs, streaming TV online and watching ‘On demand’, but not including using the TV for games consoles)
None | <1h | 1-2h | 2-3h | 3-4h |4h or more</td><td>On a weekday/weekend, how much TOTAL TIME does your child usually spend in front of a screen for leisure purposes (e.g. TV, streaming, gaming, using social media, using the internet)?

<1h | 1-2h | 2-3h | 3-4h |4-5h | 5h or more</td></tr>
<tr><td>On a weekday/weekend, how many hours does your child spend using a computer or laptop?

None | <1h | 1-2h | 2-3h | 3-4h |4h or more</td><td></td></tr>
<tr><td>On a weekday/weekend, how many hours does your child spend using a games console (e.g., XBox, Wii, PlayStation, handheld game device, etc.)?
None | <1h | 1-2h | 2-3h | 3-4h |4h or more</td><td></td></tr>
<tr><td>On a weekday/weekend, how many hours does your child spend using a smartphone (not for talking or texting) or tablet (iPad etc.)?
None | <1h | 1-2h | 2-3h | 3-4h |4h or more</td><td></td></tr>
<tr><td>On a weekday/weekend, how many hours does your child spend multi-screen-viewing (multi-screen-viewing is when you are using two or more devices at once, e.g. TV & laptop at the same time)?
None | <1h | 1-2h | 2-3h | 3-4h |4h or more</td><td></td></tr>
<tr><td colspan="2">TV-viewing time (asked for weekdays and weekends separately)</td></tr>
<tr><td>On a weekday/weekend, how many hours does your child spend watching programmes on TV (including DVDs, streaming TV online and watching ‘On demand’, but not including using the TV for games consoles)
None | <1h | 1-2h | 2-3h | 3-4h |4h or more</td><td>On a weekday/weekend, ow much of this time does your child just spend watching TV (this includes streaming on any device)?

<1h | 1-2h | 2-3h | 3-4h |4-5h | 5h or more</td></tr>
<tr><td colspan="2">Total screen-viewing time for schoolwork (asked for weekdays only)</td></tr>
<tr><td></td><td>On a weekday, how much TOTAL TIME does your child usually spend in front of a screen doing school work? (this includes online learning and homework)
<1h | 1-2h | 2-3h | 3-4h |4-5h | 5h or more</td></tr>
</table>

Table S2: Summary of missing data at age 10-11 years

	Pre-COVID-19 N=1296		Post 2020/21 lockdown N=393	
	**N**	**%**	**N**	**%**
Gender	0	0%	4	1%
Household education	105	8%	6	2%
**Screen-viewing**				
Weekday TV	226	17%	34	9%
Weekday leisure	229	18%	34	9%
Weekday schoolwork	-	-	34	9%
Weekend TV	227	18%	34	9%
Weekend leisure	241	19%	34	9%
**Devices**				
PC/Laptop	28	2%	4	1%
Phone/Tablet	16	1%	4	1%
Games console	18	1%	4	1%

- indicates data was not collected at this time point

Table S3: Time spent in screen viewing (mins) by child gender and household education

	Age 8-9		Age 10-11			
	Pre-COVID-19		Pre-COVID-19		Post 2020/21 lockdown	
	Mean (SD)	Median (IQR)	Mean (SD)	Median (IQR)	Mean (SD)	Median (IQR)
**All children**						
Weekday TV[1]	65 (48)	30 (60)	62 (40)	90 (60)	99 (63)	90 (60)
Weekday total leisure[2]	100 (68)	90 (60)	147 (92)	120 (90)	144 (77)	150 (60)
Weekday schoolwork[3]	-	-	-	-	65 (67)	30 (60)
Weekend TV[1]	122 (65)	90 (60)	82 (57)	90 (0)	135 (67)	150 (60)
Weekend total leisure[2]	192 (109)	180 (120)	203 (112)	180 (150)	194 (73)	210 (60)
**Child Gender**						
Boys						
Weekday TV[1]	58 (41)	30 (60)	60 (38)	30 (60)	94 (61)	90 (60)
Weekday total leisure[2]	103 (67)	90 (90)	152 (95)	120 (90)	147 (81)	150 (120)
Weekday schoolwork[3]	-	-	-	-	61 (65)	30 (60)
Weekend TV[1]	111 (62)	90 (60)	79 (55)	90 (90)	131 (66)	150 (60)
Weekend total leisure[2]	204 (111)	180 (150)	211 (110)	210 (120)	199 (75)	210 (120)
Girls						
Weekday TV[1]	71 (52)	90 (60)	63 (41)	90 (60)	106 (65)	90 (30)
Weekday total leisure[2]	98 (69)	90 (60)	143 (89)	120 (90)	141 (73)	90 (60)
Weekday schoolwork[3]	-	-	-	-	69 (69)	30 (60)
Weekend TV[1]	131 (67)	150 (60)	85 (58)	90 (0)	141 (67)	150 (120)
Weekend total leisure[2]	183 (106)	180 (120)	196 (113)	180 (150)	188 (70)	210 (60)
**Household education**						
Up to A Level equivalent						
Weekday TV[1]	74 (51)	90 (60)	68 (41)	90 (60)	122 (71)	90 (60)
Weekday total leisure[2]	112 (76)	90 (90)	167 (98)	150 (120)	168 (86)	150 (120)
Weekday schoolwork[3]	-	-	-	-	81 (79)	30 (60)
Weekend TV[1]	129 (69)	150 (60)	86 (59)	90 (0)	153 (71)	150 (120)
Weekend total leisure[2]	200 (118)	180 (150)	217 (121)	210 (135)	211 (74)	210 (120)
Degree equivalent or higher						
Weekday TV[1]	57 (44)	30 (60)	59 (38)	30 (60)	88 (55)	90 (60)
Weekday total leisure[2]	91 (60)	90 (60)	130 (82)	120 (60)	131 (69)	90 (60)
Weekday schoolwork[3]	-	-	-	-	57 (59)	30 (60)
Weekend TV[1]	116 (62)	90 (60)	79 (54)	90 (90)	127 (63)	90 (60)
Weekend total leisure[2]	186 (101)	180 (120)	191 (101)	180 (120)	185 (70)	210 (60)

[1] TV viewing includes on-demand and streaming to any device.

[2] Total screen-viewing includes TV viewing.

[3] data for weekday schoolwork was only collected post COVID-19.

- indicates data was not collected at this time point

Table S4: Access to different devices at age 10-11 years, pre-COVID-19 and post 2020/21 lockdown

	Pre-COVID-19<br>N=1256	Post 2020/21 lockdown<br>N=389
PC/Laptop	78%	72%
Phone/Tablet	96%	87%
Games console	79%	71%
Any device	99%	94%

Note, games consoles included handheld devices

Table S5: Modelled difference in child weekday and weekend total leisure time screen viewing between pre-COVID-19 and post 2020/21 lockdown, adjusting for number of types of devices

	Difference between pre-COVID-19 and post 2020/21 lockdown		Interaction effect	
	Relative Risk (95% CI)	*P*-value	Relative Risk (95% CI)	*P*-value
**Weekday total leisure screen-viewing[1] (min)**		**N=1395**		
No interaction	1.12 (1.10 to 1.05)	<0.0005		
Gender interaction				
Boys	1.11 (1.08 to 1.14)	<0.0005		
Girls	1.14 (1.11 to 1.17)	<0.0005	1.02 (1.00 to 1.04)[3]	0.020
Education[2] interaction				
up to A level	1.11 (1.09 to 1.14)	<0.0005	1.02 (1.00 to 1.04)[4]	0.084
Degree or higher	1.13 (1.10 to 1.16)	<0.0005		
**Weekend total leisure screen-viewing[1] (min)**		**N=1382**		
No interaction	1.09 (1.07 to 1.11)	<0.0005		
Gender interaction				
Boys	1.07 (1.02 to 1.07)	<0.0005		
Girls	1.11 (1.08 to 1.13)	<0.0005	1.04 (1.02 to 1.06)[3]	<0.0005
Education[2] interaction				
up to A level	1.07 (1.05 to 1.11)	<0.0005	1.03 (1.01 to 1.05)[4]	0.001
Degree or higher	1.11 (1.08 to 1.13)	<0.0005		

[1] total screen-viewing includes TV viewing.

[2] or equivalent; A level is exam at age 18

[3] RR for girls compared to boys

[4] RR for up to A level compared to degree or higher.

Models are adjusted for age, gender, household education, seasonality and COVID-19 restrictions.

Table S6: Post 2020/21 lockdown demographic and screen-viewing summaries of interview and focus group participants compared to quantitative sample

	Parent Interviews			Child Focus groups			Quantitative sample		
	N	%	Mean (SD)	N	%	Mean (SD)	N	%	Mean (SD)
**Parent Gender**									
Male	0	0%		9	19%		91	24%	
Female	21	100%		37	79%		295	76%	
Other	0	0%		1	2%		1	<0.5%	
**Parent Age (years)**									
<39	3	14%		10	21%		117	30%	
40+	18	86%		37	79%		270	70%	
**Child gender**									
Male	10	48%		26	55%		198	51%	
Female	11	52%		21	45%		191	49%	
Other	0	0%		0	0%		2	0.5%	
**Household education**									
Up to A level[1]	5	24%		19	40%		136	35%	
Degree or higher[1]	16	76%		18	60%		251	65%	
**Screen-viewing (mins)**									
Weekday TV[2]			69 (35)			97 (57)			99 (63)
Weekday leisure			120 (63)			153 (85)			144 (77)
Weekend TV[2]			108 (70)			135 (57)			135 (67)
Weekend leisure			192 (85)			204 (74)			194 (73)

[1] or equivalent; A level is exam at age 18

[2] TV viewing includes on-demand and streaming to any device
