# Supplementary material for: Screen-viewing behaviours of children before and after the 2020–21 COVID-19 lockdowns in the UK: a mixed methods study
Source: BMC Public Health. 2023 Jan 17;23:116. doi: 10.1186/s12889-023-14976-6 (PMC9843116; doi:10.1186/s12889-023-14976-6)
Supplement: Supplementary file 2 — Additional file 2: Supplementary File. Interview and focus group topic guides. [file 12889_2023_14976_MOESM2_ESM.pdf]

Screen-viewing in children before and after the 2020-2021 COVID-19 lockdowns in the UK: a mixed methods study

Authors: Ruth Salway, Robert Walker, Kate Sansum, Danielle House, Lydia Emm-Collison, Byron Tibbitts, Tom Reid, Katie Breheny, Sarah Churchward, Joanna G Williams, Frank de Vocht, William Hollingworth, Charlie Foster and Russell Jago

Supplementary File: Interview and focus group topic guides

This file includes the guides for: A) Parent interviews; and B) Child focus groups.

Please note that this study was drawn from a larger project (Active-6) that is exploring the impact of the COVID-19 pandemic on children and their parents' physical activity and screen-viewing behaviour. Subsequently, the interview/focus group guides below reflect this. Due to the volume and nature of information gained during these interviews, physical activity-related information was published separately to screen-viewing. Please see this paper for discussion related to physical activity:

Walker R, House D, Emm-Collison L, Salway R, Tibbitts B, Sansum K, et al. A multi-perspective qualitative exploration of the reasons for changes in the physical activity among 10–11-year-old children following the easing of the COVID-19 lockdown in the UK in 2021. Int J Behav Nut Phys Act. 2022;19(1),1-13.

A) **Parent interview guide**

Introduction and consent

Thank you for agreeing to take part in this interview, your views and opinions are really important to us.

In the interview today, I would like to talk about three main points:

1. You and your child's physical activity patterns and how these may have been impacted by COVID-19.
2. Factors that you feel you have caused any changes
3. Whether you think there is anything that can be done to help support you and your child's physical activity

We are really interested in your honest opinions, we are not here to judge you, and we do not want you to feel like you should answer any of the questions in a certain way. There are no right or wrong answers, and as much detail you can give on the topics as possible is really appreciated.

Before we get started, I'd like to go over some important information regarding the interview and your data:

- I will be **recording the conversation** to help us remember what you said
- You can ask for the recording to be stopped at any time
- After we have written a report about all the opinions we have heard from the parents taking part, the recordings will be destroyed
- We will also change any names or identifying information so none of the information that is written down and recorded can be connected to you in any way
- Please remember that you can interrupt the interview at any point if you need to.
- If you do not want to answer a question please say so.
- Are you comfortable to proceed with the interview?

If the parent says yes the recording will start. As noted on the information sheet the interview will be recorded.

- For the recording, can you please confirm that you have been given information explaining about the study and that you understand what this project is about?
- Can you please confirm that you have had the opportunity to ask questions and discuss this study?
- Can you please confirm that you are aware that the interview data will be stored anonymously and securely for 20 years?
- Can you please confirm that you are aware that you are free to withdraw your data from the study at any time up to three weeks after this interview has taken place **[X DATE]**, and that you do not need to give a reason for withdrawing?
- And finally, can you please confirm that you are happy to take part in the interview?

Icebreakers

1. Discussion of physical activity definition (4 domains)
    a. Leisure-time
    b. Transport
    c. Household
    d. Occupational
2. Can you tell us what your child in Year 6's favourite physical activity/physically active thing to do is and why do you think this is their favourite?
3. Can you describe your favourite physical activity/physically active thing to do is and why do you think this is their favourite?

Changes in PA and device/screen time over COVID pandemic

Pre-lockdown

1. How would you describe your activity levels before the first lockdown?
    - Did you often use active modes of transport?
    - Were you part of any active clubs or have any active hobbies?
    - Are you very active around the house?
    - If you are employed, is your work active?
2. How active was your child before the first lockdown?
    - Did your child use active modes of transport to school?
    - Did they participate in any active clubs or hobbies?
    - If you feel you can answer, were they active during playtimes?

First school closure/lockdown 1 (March 2020)

3. Did your child attend school at this time? (i.e. parents were key workers)
    a. If so, to what extent do you feel this influenced their activity levels?
4. To what extent do you feel the first lockdown/school closure influence your activity levels?
5. How about your child's?
6. What were the key factors that influenced any changes to your/your child's PA at this time?
   Pool of prompts (exact prompts used will be guided by the contact's role/previous discussions):

**Policy**	
1. To what extent do you feel that any policy/legal restrictions associated with COVID-19 effected your/your child's (i.e. lockdowns, social distancing)?	□
**Environment**	
2. To what extent did you/your child's opportunities to be physically active outside of school change at this time?<br>a. How did you feel about these changes?	□
3. How do you feel any changes to school or school curriculum PE influence your child's activity levels?<br>a. How did you feel about these changes?<br>b. Did you feel supported in doing PE from home?	□
**Strategies to promote PA**	
4. To what extent did you feel supported and encouraged by the school or other organisations to be physically active at this time?	□
**Attitudes**	
5. Can describe your attitude and thoughts towards physical activity at this time? Had it changed?	□
6. To what extent do you feel that the school's attitude towards PE and other physical activities changed at this time?	□
7. To what extent do you feel that being less or being more active was the norm during lockdowns, or did it stay the same? Was this the same for both child and parent?	□
**Social**	
8. To what extent do you feel your child's active play with their friends changed at this time?	□
9. To what extent did social distancing and not being able to see other people influence your/your child's physical activity?	□
**Motivation**	
10. To what extent do you feel that your/child's motivation for physical activity changed at this time?	□
11. Have you noticed changes related to confidence in your ability to do PA in your child? If so, how did it change?	□
Emotion	
12. Thinking back to how you felt at this time, to what extent did your/your child's feelings influence your activity levels? For example, was lockdown 1 a novelty that made you want to be more active or were you very worried and did not want to leave your house?	□

First return to school (Autumn 2020)

7. Did your child attend school this time? If so, what had changed?
8. To what extent did the lifting of the lockdown and return to school influence your physical activity levels?
9. How about your child's?
10. What were the key factors that influenced any changes to your/your child's activity at this time?

Second school closure (January – March 2021)

11. Did your child attend school at all at this time?
    a. If so, what had changed?
    b. To what extent do you feel this influenced their activity levels?
12. Thinking back to the first lockdown/school closure, to what extent did your/your child's activity levels differ in the second lockdown/closure compared to the first?
13. Can you please describe any differences between this lockdown/school closure and the first?

Second (most recent) reopening of schools (April – July 2021)

14. To what extent did the most recent return to school influence your activity levels?
15. How about your child's?
16. What were the key factors that influenced any changes to your/your child's PA at this time?

Other factors influencing PA

17. Are there any other factors that we haven't discussed that you think have influenced your/your child's activity levels over the course of the pandemic so far?

Electronic device use/screen time

18. How has your child's use of electronic devices changed along the way – total time and how they use them (i.e. for school work/socializing/gaming or watching things)?

Current solution-focused ideas for themselves and their child

19. Evidence from government reports and other research suggests that physical activity has decreased since the pandemic started. To what extent does this match up with your experiences?
20. Are there any barriers that are ***currently*** preventing you/your child from being more active?
    a. How and who could support you to remove these barriers?
21. Is there anything that could help to boost your/your child's motivation to be active?
22. Is anything needed to help enable you/your child to do more of the active things you/they wish to do?
    a. Where/when/how should it be available?

b. How could the school support it?
c. How could local communities/local government support it?
d. How could national government support?

Closing statement

- Is there anything else you'd like to tell us about the things we talked about today?
- Do you have any questions for me?
- We appreciate you sharing your thoughts and opinions with us!

B) Student focus group guide

Introduction (10-15 minutes)

- Thank children for participating in the ACTIVE-6 project
- Introduce myself
- Explain why this project is important and what it means for them

Active travel (10 minutes)

1. Who here has ever walked, biked, or scooted to school before?
2. Who has changed how they travel to school since coming back after lockdown?
3. If you could choose anyway to travel to school, what would you choose? Why? What is stops you from doing that?

Physical activity during school time (15 minutes)

School PE

4. What is everyone's favourite thing to do in PE? What did you like about it?
5. How did everyone feel about PE before, during, and after?
6. Has your feelings changed toward PE since after the lockdowns?

Breaktime

7. Who likes playing in the playground? What kind of things do you do?
8. Did anyone miss not being able to play in the playground when schools were closed?
9. How was it going back into the playground after lockdown?
10. Did it feel different?

Physical activity outside of/after school (15 minutes)

Activity: Ask children to draw themselves doing the physical activity they do the most at home. For example, playing with friends and going on walks with family. Ask them to add words around it to describe how it makes them feel. Children will have 2 minutes to draw their best stick figure-style drawing.

Active clubs

11. Who did an active club, such as football or swimming, before coronavirus? **(SHORT)**
12. After schools were closed, what happened to your active club? Did it continue online or was it cancelled?
13. What was it like going back to active clubs after lockdown?
14. Did anybody choose not to go back to their active club because of COVID?
15. Who has started an active club after coming back to school? What made you want to start one?

At home/screen time

16. How did everyone spend their free time during lockdown?
17. Was this different to what you used to do before lockdown?
18. Are you still spending your free time that way?

19. Does anyone think their parents rules about screen time changed?

Closing statement and questions

- Thank all the children and tell them they have been a big help with our research
- Overall, is there anything anyone would like to talk about or mention that we haven't already discussed?
- Are there any questions?
- Say goodbye and end focus group
